# Supplementary material for: Melanoma Cell Reprogramming and Awakening of Antitumor Immunity as a Fingerprint of Hyper-Harmonized Hydroxylated Fullerene Water Complex (3HFWC) and Hyperpolarized Light Application In Vivo
Source: Nanomaterials (Basel). 2023 Jan 17;13(3):372. doi: 10.3390/nano13030372 (PMC9918970; doi:10.3390/nano13030372)
Supplement: Supplementary file 1 [file nanomaterials-13-00372-s001.zip › nanomaterials-2097167-supplementary.pdf]

Supporting information

Melanoma Cell Reprogramming and Awakening of Antitumor Immunity as a Fingerprint of Hyper-Harmonized Hydroxylated Fullerene Water Complex (3HFWC) and Hyperpolarized Light Application *In Vivo*

Milica Markelić [1,*,†], Marija Mojić [2,†], Dijana Bovan [2], Sanja Jelača [2], Zorana Jović [3], Milica Purić [4], Djuro Koruga [3], Sanja Mijatović [2], Danijela Maksimović-Ivanić [2,*]

[1] Faculty of Biology, University of Belgrade, 11000 Belgrade, Serbia
[2] Institute for Biological Research “Siniša Stanković”- National Institute of the Republic of Serbia, University of Belgrade, 11060 Belgrade, Serbia; marija.mojic@gmail.com (M.M.); dijana.draca@ibiss.bg.ac.rs (D.B.); sanjajelaca93@gmail.com (S.J.); sanjamama@ibiss.bg.ac.rs (S.M.)
[3] TFT NanoCenter, 11050 Belgrade, Serbia; zorana.jovic@tftnanocenter.rs (Z.J.); djuro.koruga@gmail.com (D.K.)
[4] ZeptoHyperTech, 11070 Belgrade, Serbia; milica.vuksanovic@zepterhypertech.com
* Correspondence: milica.markelic@bio.bg.ac.rs (M.M.); nelamax@ibiss.bg.ac.rs (D.M.-I.)
† These authors contributed equally to this work.

List of contents

Figure S1. Representative micrographs of tumor necrosis area and TUNEL detection of apoptotic cells in the melanoma tissue from HPL, 3HFWC and 3HFWC+HPL-treated mice with syngeneic melanoma.

Figure S2. Representative micrographs of the most significant histopathological alterations in the liver and kidney cortex of HPL, 3HFWC and 3HFWC+HPL-treated mice with syngeneic melanoma.

Table S1. Biochemical urine parameters of HPL, 3HFWC and 3HFWC+HPL-treated mice with syngeneic melanoma

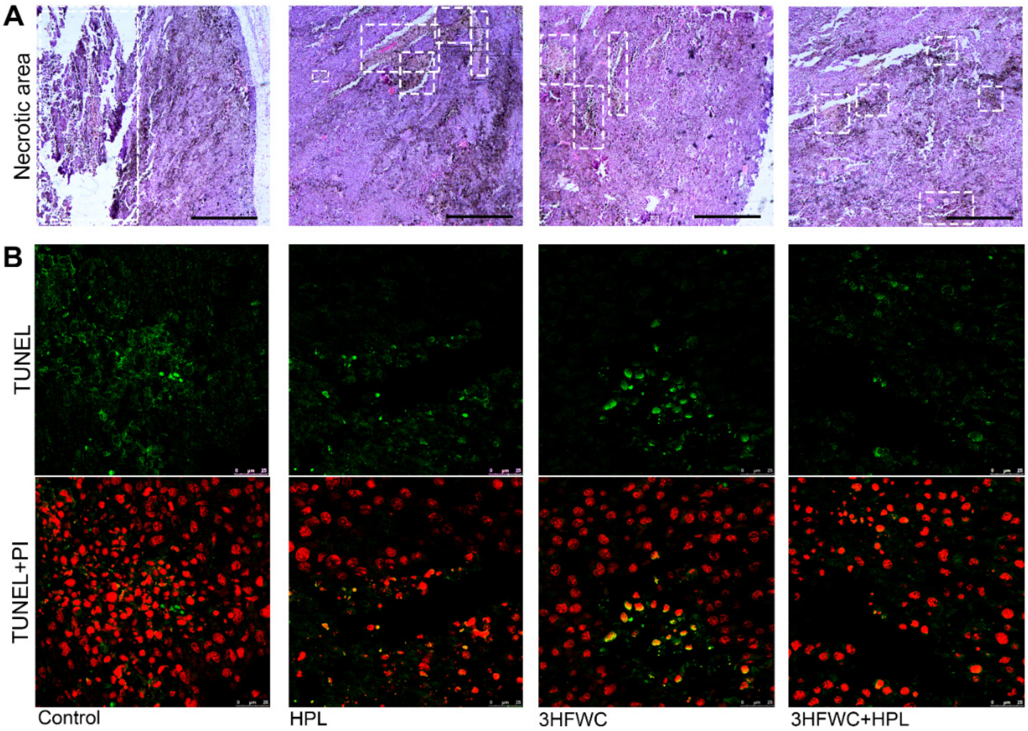

Figure S1. Representative micrographs of **A**) tumor necrosis area (squares), HE staining (scale bar and orig. magnification – 500 μm, × 5); and **B**) TUNEL detection of apoptotic cells (green signal, alone and merged with red PI signal of nuclei; (scale bar and orig. magnification – 25 μm, × 63); in the melanoma tissue from HPL, 3HFWC and 3HFWC+HPL-treated mice with syngeneic melanoma.

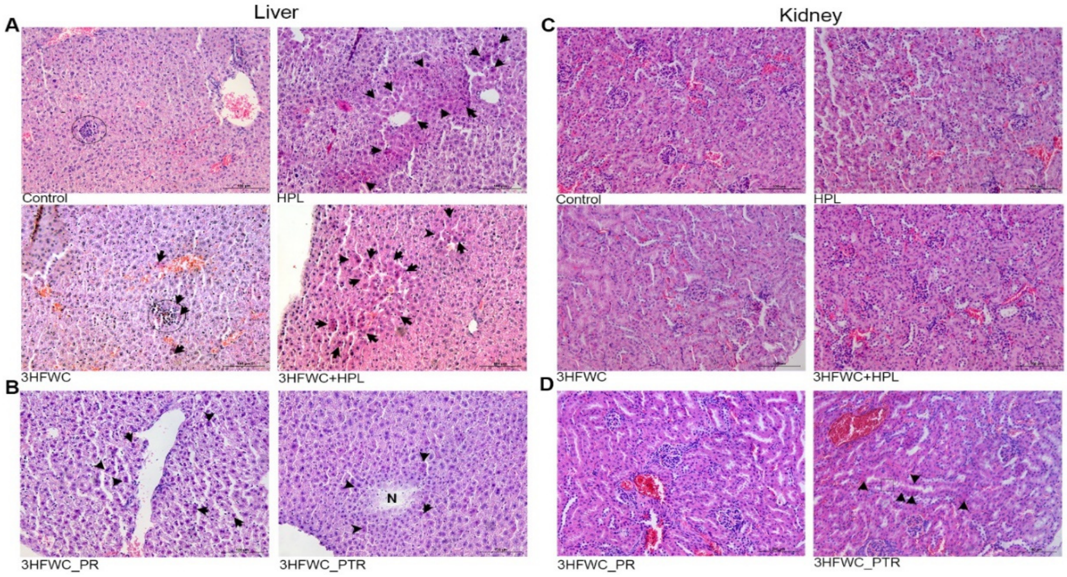

Figure S2. Representative micrographs of the most significant histopathological alterations in the (**A**, **B**) liver and (**C**, **D**) kidney cortex of HPL, 3HFWC and 3HFWC+HPL-treated mice with syngeneic melanoma. A) Liver tissue of animals treated after the melanoma induction; **B**) liver tissue of animals treated with 3HFWC in prophylactic (PR) and combined (prophylactic+therapeutic, PTR) regimen; **C**) Kidney tissue of animals treated after the melanoma induction; **D**) kidney tissue of animals treated with 3HFWC in PR and PTR. Encircled area – immune cells granulomas; arrowheads – eosinophilic, apoptotic cells; N – necrotic area; square – discontinuation of tubular epithelium. HE staining; scale bar and orig. magnification – 100 μm, × 10

Table S1. Biochemical urine parameters of HPL, 3HFWC and 3HFWC+HPL-treated mice with syngeneic melanoma.

	**Control**	**3HFWC**	**HPL**	**3HFWC + HPL**
Spec. gravity	1027 ± 2.7	1028 ± 2.7	1028. 3 ± 2.9	1028, 3 ± 2.9
pH	5 ± 0	5.2 ± 0.4	5.7 ± 0.6	5 ± 0
Glucose (mmol/L)	5.5 ± 0	5.5 ± 0	5.5 ± 0	5.5 ± 0
Nitrite	negative	negative	negative	negative
Protein (mg/100ml)	40 ± 34.6	76 ± 40.4	10 ± 0	16.7 ± 11.5
Ketones (mg/100ml)	8 ± 2.7	8.3 ± 2.8	8.3 ± 2.9	8.3 ± 2.9
Urobilinogen (mg/100ml)	4.2 ± 2.5	3.0 ± 3.0	4.0 ± 0	2.0 ± 1.7
Bilirubin (mg/100ml)	0.8 ± 0.3	0.4 ± 0.2	0.5 ± 0	0.5 ± 0
Blood (RBC/μl)	negative	negative	negative	negative
Leukocytes (WBC/μl)	negative	negative	negative	negative
